# Evaluation of hypoxia in an experimental rat tumour model by [^18^F]Fluoromisonidazole PET and immunohistochemistry

**DOI:** 10.1038/sj.bjc.6602219

**Published:** 2004-11-02

**Authors:** L Dubois, W Landuyt, K Haustermans, P Dupont, G Bormans, P Vermaelen, P Flamen, E Verbeken, L Mortelmans

**Affiliations:** 1 Department of Nuclear Medicine, University Hospital Gasthuisberg and KU Leuven, Herestraat 49, 3000 Leuven, Belgium; 2 Lab Experimental Radiobiology/LEO, University Hospital Gasthuisberg and KU Leuven, Herestraat 49, 3000 Leuven, Belgium; 3 Lab Radiopharmaceutical Chemistry, University Hospital Gasthuisberg and KU Leuven, Herestraat 49, 3000 Leuven, Belgium; 4 Morphology and Molecular Pathology, University Hospital Gasthuisberg and KU Leuven, Herestraat 49, 3000 Leuven, Belgium

**Keywords:** hypoxia, tumour, [^18^F]FMISO-PET, pimonidazole, CA IX

## Abstract

This study aimed to evaluate tumour hypoxia by comparing [^18^F]Fluoromisonidazole uptake measured using positron emission tomography ([^18^F]FMISO-PET) with immunohistochemical (IHC) staining techniques. Syngeneic rhabdomyosarcoma (R1) tumour pieces were transplanted subcutaneously in the flanks of WAG/Rij rats. Tumours were analysed at volumes between 0.9 and 7.3 cm^3^. Hypoxic volumes were defined using a 3D region of interest on 2 h postinjection [^18^F]FMISO-PET images, applying different thresholds (1.2–3.0). Monoclonal antibodies to pimonidazole (PIMO) and carbonic anhydrase IX (CA IX), exogenous and endogenous markers of hypoxia, respectively, were used for IHC staining. Marker-positive fractions were microscopically measured for each tumour, and hypoxic volumes were calculated. A heterogeneous distribution of hypoxia was observed both with histology and [^18^F]FMISO autoradiography. A statistically significant correlation (*P*<0.05) was obtained between the hypoxic volumes defined with [^18^F]FMISO-PET and the volumes derived from the PIMO-stained tumour sections (*r*=0.9066; *P*=0.0001), regardless of the selected threshold between 1.4 and 2.2. A similar observation was made with the CA IX staining (*r*=0.8636; *P*=0.0006). The relationship found between [^18^F]FMISO-PET and PIMO- and additionally CA IX-derived hypoxic volumes in rat rhabdomyosarcomas indicates the value of the noninvasive imaging method to measure hypoxia in whole tumours.

As a result of rapid growth and insufficient blood supply, solid tumours demonstrate hypoxia and necrosis in heterogeneous spread regions (Chapman, 1984; Vaupel *et al*, 1989). The presence of hypoxia is a major cause of resistance to cancer treatment. Hypoxia has been shown to be an independent predictor of poor progression-free survival in several types of cancer (Brizel *et al*, 1997; Nordsmark and Overgaard, 2000; Fyles *et al*, 2002; Kaanders *et al*, 2002). Treatments to overcome the effect from a hypoxic environment are being evaluated (Brown, 1999; Wouters *et al*, 2003). Therefore, the assessment of tumour oxygenation will be valuable to guide treatment in individual patients.

To date, various methods are available to measure tumour oxygenation, including polarographic oxygen sensors (Vaupel *et al*, 1991), luminescence-based optical sensors (Bussink *et al*, 2000) and antibody-based detection of exogenous (Haustermans *et al*, 2000; Raleigh *et al*, 2001; Koch, 2002) and endogenous markers of hypoxia (Wykoff *et al*, 2000; Janssen *et al*, 2002; Airley *et al*, 2003). At this time, only the polarographic oxygen-sensor and the exogenous hypoxia marker pimonidazole (PIMO) can be considered as ‘standard’. These methods may have limitations in clinical settings due to the invasive character of the method, the limited accessibility of the tumours and the possibility of sampling errors. Therefore, methods for noninvasive detection of hypoxia are under continued development. These methods include magnetic resonance imaging techniques (Robinson *et al*, 1998; Mason *et al*, 1999; Landuyt *et al*, 2001) and the detection of sensitiser adducts with SPECT and PET (Chapman *et al*, 1998; Ballinger, 2001; Hoebers *et al*, 2002; Van de Wiele *et al*, 2003).

The most widely used PET radiotracer for imaging tumour hypoxia is [^18^F]Fluoromisonidazole ([^18^F]FMISO). The use of labelled 2-nitroimidazole compounds is based on studies with [^3^H]misonidazole binding in several human tumour types (Urtasun *et al*, 1986). Although the [^3^H]misonidazole approach had limited clinical utility, it was the start of the development of noninvasive hypoxia markers based on 2-nitroimidazoles. The selective binding of [^18^F]FMISO to hypoxic cells has been demonstrated *in vitro* as well as preclinically *in vivo* (Rasey *et al*, 1989; Koh *et al*, 1992).

The present study reports on advances towards the *in vivo* validation of [^18^F]FMISO for the evaluation of tumour hypoxia. This was carried out by comparing [^18^F]FMISO-PET measurements with immunohistochemical analysis using PIMO, another nitroimidazole which is established as an exogenous intracellular hypoxia marker. To obtain potential complementary information, comparison was made with an endogenous transmembranous hypoxia marker carbonic anhydrase IX (CA IX). To our knowledge, it is the first time that [^18^F]FMISO uptake measured with PET was compared with PIMO, and additionally with CA IX staining, to evaluate hypoxic volumes in tumours.

## MATERIALS AND METHODS

### Animals and tumour model

Male adult WAG/Rij rats with an average body weight of 300 g were used. Each rat was subcutaneously implanted under anaesthetics with syngeneic rhabdomyosarcomas (1-mm^3^ R1 tumours) in the lateral thorax or in the abdominal flank. After 12 days, when tumours reached the predetermined range of volumes, PET measurements were carried out during a 2-week follow-up. Each day, tumours were measured, using a Vernier calliper, in three orthogonal tumour diameters *A*, *B* and *C*, each corrected for the thickness of the skin. Volumes were calculated using the formula *A* × *B* × *C* × *π*/6, since the tumours grew elliptically. All animal experiments were conducted in accordance with local institutional guidelines, approved by the Animal Ethics Committee of the University ‘KU Leuven’ and procedures were according to the guidelines defined by the UKCCCR (Workman *et al*, 1998).

### Radiolabelled tracer

1-(2-nitro-imidazolyl)-3-[^18^F]-fluoro-2-propanol ([^18^F]Fluoromisonidazole; [^18^F]FMISO) was produced by the nucleophilic fluorination of 1-(2′-nitro-1′-imidazolyl)-2-*O*-tetrahydropyranyl-3-*O*-toluenesulphonylpropandiole followed by acidic hydrolysis of the protecting group, as described by Lim and Berridge (1993).

### Experimental set-up

When PET measurements were started, each rat received [^18^F]FMISO every 2 days. The rats were anaesthetised with 0.1 ml 100 g^−1^ body weight sodium pentobarbital (Nembutal; Sanofi, Belgium), injected intraperitoneally. The radiolabelled tracer was administrated via an intravenous line (Venoflux 0.4 mm G.27; Vygon, France) inserted into a lateral tail vein, flushed with heparin saline solution. During PET examinations, anaesthesia was maintained using individual rat-adapted intraperitoneal injections (10–30% of the initial dose) of the anaesthetic, as needed.

### PET imaging

Each PET examination was performed on an ECAT HR^+^ scanner (Siemens, Knoxville, TN, USA), with an axial field of view of 15 cm and a spatial resolution of 6 mm full-width at half-maximum (FWHM) at the centre of the field of view. Before positioning, four rats were placed in a custom-built, polystyrene foam that was placed in the opening of the camera. Whole-body scanning was performed with 63 axial slices, each of 2.425 mm, in a single 15-cm field of view, yielding radioactivity concentration measurements in voxels of 1.6875 × 1.6875 × 2.425 mm^3^. The [^18^F]FMISO-PET acquisition started with a 15-min transmission scan using external rods of ^68^Ge to correct for attenuation. An average of 17.02 MBq (13.38–21.24 MBq) [^18^F]FMISO was injected into a lateral tail vein. Simultaneously, a dynamic emission scanning was started for 60 min, according to the following protocol: 8 × 15 s, 4 × 30 s, 2 × 1 min, 2 × 2 min, 10 × 5 min. At 2 h postinjection (p.i.), a second dynamic emission scanning was carried out for 20 min (4 × 5 min). Preceding this second scanning, a 15-min transmission scan was performed after repositioning the rats. All images were reconstructed iteratively in a 128 × 128 × 63 matrix using attenuation factors measured by the transmission scans. The images were corrected for scatter and randoms, frame duration and decay to the start of each emission scan. The radioactivity measured in selected tissues, determined on the images, was subsequently corrected for decay towards the time of injection.

### PET image analysis

To obtain mean activity data of the blood pool, a region of interest (2D-ROI), representing a volume of 0.425 cm^3^, was drawn at the efflux area of the heart on the 2 h p.i. [^18^F]FMISO images. The efflux area of the heart was defined with the aid of the early [^18^F]FMISO perfusion images (summation of 1–4 min of the first dynamic acquisition). Normal tissue evaluation was carried out using 2D-ROI analysis of the lung, muscle (cf Koh *et al*, 1992) and a body area 15 mm above the heart (cf Bentzen *et al*, 2000), and normal tissue to heart mean activity ratios were calculated. To assess the volume of [^18^F]FMISO uptake in tumours, different absolute thresholds were used, ranging from 1.2 to 3.0. A 3D-ROI was defined on the 2 h p.i. [^18^F]FMISO images to select all the voxels with activity higher than a selected absolute threshold.

### Pimonidazole administration

During the 2-week follow-up, a rat was killed daily to enable immunohistochemical analyses of tumour volumes ranging between 0.9 and 7.3 cm^3^. A total of 11 tumours were selected to cover the full volume range. Each rat received 0.1 ml 100 g^−1^ body weight of pimonidazole hydrochloride (Hypoxyprobe-1; NPI, Belmont, MA, USA) intraperitoneal prior to the start of the second dynamic emission scanning. At 1 h p.i. of PIMO (i.e. 40 min after the end of scanning), the rat was killed and the tumours were rapidly excised for immunohistochemical processing.

### Immunohistochemistry

Excised tumours were fixed in neutral-buffered formalin and axial 2 mm sections were made (*n*=3–6, depending on tumour size), according to the PET plane separation thickness, prior to embedding in paraffin. Slices (5 *μ*m) from each axial section were deparaffinised with toluene and rehydrated by treatment with a series of alcohol and water mixtures and finally with water. To quench endogenous peroxidase, the tissue sections were exposed for 30 min to 0.3% hydrogen peroxide absolute methanol. Microwave heating (4 min 500 W, 20 min defrost and 15 min RT) was used in the presence of Tris EDTA buffer (0.01 M pH=9.0) to achieve antigen retrieval prior to the application of the primary monoclonal antibody. Phosphate-buffered saline (PBS 0.1 M pH=7.3) plus Tween 20 (polyoxyethylene sorbitan monolaurate) were used to wash slides between two steps. The sections were incubated in anti-PIMO MAb (1 : 100 dilution in PBS) for 30 min. Secondary incubation with peroxidase-labelled anti-mouse Envision (DAKO Corporation, Carpinteria, CA, USA) was applied also for 30 min. Bound peroxidase was developed using 0.033% hydrogen peroxide in 10% diaminobenzidine (DAB; DAKO Corporation, Carpinteria, CA, USA) for 7 min. After washing in distilled water, the sections were counterstained with haematoxylin for 1 min, dehydrated and mounted.

Sections contiguous to those stained for PIMO binding were immunostained for the presence of CA IX in the same manner that was used for PIMO adducts. M75 MAb (Bayer, USA; Pastorekova *et al*, 1992) was used as primary antibody (1 : 25 dilution in PBS). Substitution of the primary antibody with PBS-Tween was used as a negative control for both antibodies.

Immunostained sections (*n*=50 both for PIMO and for CA IX) of the selected tumours (*n*=11) were viewed by means of a Zeiss Axioskop 40 FL microscope (Carl Zeiss, Inc., Thornwood, NY, USA). Each section was evaluated independently by two investigators (LD and WL) and scored by means of moving a ‘10 × 10’ grid-incorporated ocular, superimposed on the image, at a total magnification of × 200. Five hit-points in every grid, lying on the intersection of the grid lines, were evaluated for positive PIMO and CA IX staining in contiguous sections (Weibel, 1981). For each tumour, depending on the size, 350–1260 hit-points were evaluated for PIMO and CA IX separately, and the respective fraction staining positive was calculated. Care was taken that the hit-points covered the entire tumour area.

### Autoradiography

In two tumour-bearing rats, an autoradiography experiment was carried out. The experimental set-up was the same as described above, but without carrying out the actual PET imaging. An average of 84.75 MBq (78.13–91.37 MBq) [^18^F]FMISO was injected intravenous via the tail vein. At 150 min p.i., the rats were killed, the tumours quickly removed and immediately frozen in 2-methylbutane (cooled to −25°C with liquid nitrogen). The tumours were cut with a Leica CM 3050 cryotome (Germany) in 14 *μ*m-thick slices at each sectional plane of 2 mm and mounted on microscope slides. The slices were immediately air dried at 50°C and exposed overnight to a high-performance storage phosphor screen (Packard, Meriden, USA), which was scanned in a Phosphor Imager Scanner (Packard Cyclone TM, Meriden, USA). The resolution of the images expressed in pixel size was 42 × 42 *μ*m (600 d.p.i.).

### Statistics

All statistical analysis and graphs are performed with Statistica (data analysis software system), version 6.0. (Statsoft, Inc., 2001, Tulsa, OK, USA). Correlation among the variables was analysed using the ‘Spearman Rank Order Correlation’ and additionally the relationships were evaluated by linear regression. A ‘Student's *t*-test’ was used to determine the statistical significance of differences between two independent groups of variables. For all tests, a *P* < 0.05 was considered significant.

## RESULTS

### Immunohistochemical analysis of tumours: PIMO, and CA IX, positive fraction

**Figure 1:**
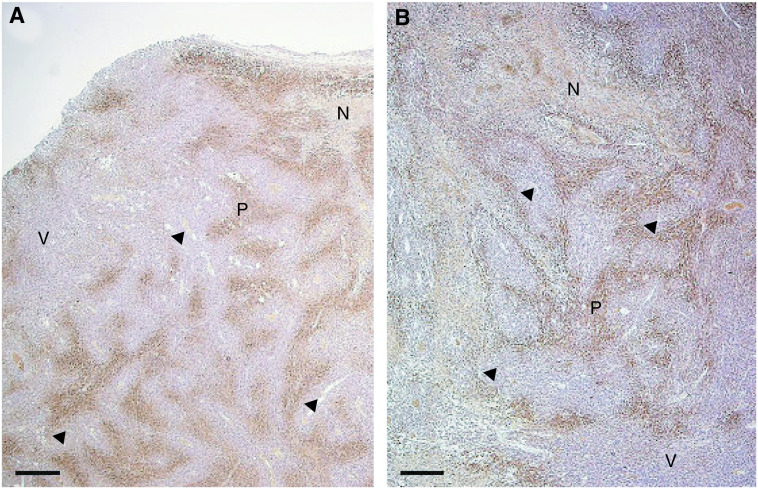
Pimonidazole staining photographs (made with Carl Zeiss KS100 Software). (**A**) Peripheral view. (**B**) Central view. Both slices are shown on a magnification × 25. Scale bar is 40 *μ*m. Abbreviations: N=necrosis, V=viable, well-oxygenated tumour tissue, P=PIMO-positive staining and the arrow indicates a blood vessel.

Independent of the tumour size (range: 0.9–7.3 cm^3^), PIMO-positive staining areas were seen in all tumour sections and they were heterogeneously distributed along the sections, as shown in Figure 1

. Localisation of the MAb stain was always at a distance (several cell layers) from a blood vessel, most often near an area of necrosis, in peripheral as well as central parts of the sections. Similar heterogeneous staining areas were found in CA IX-stained sections.

After microscopic grid-based analysis of the immunostained sections, the staining-positive fraction was calculated for each tumour (see Table 1

**Table 1:** Tumour volumes, PIMO-positive and CA IX-positive fractions, with their mean value and standard deviation and PIMO-positive and CA IX-positive volumes for 11 rhabdomyosarcoma (R1) tumours transplanted in WAG/Rij rats

***n*=11**	**Tumour volume (cm^3^)**	**PIMO-positive fraction**	**CA IX-positive fraction**	**PIMO-positive volume (cm^3^)**	**CA IX-positive volume (cm^3^)**
	0.89	0.197	0.195	0.175	0.174
	0.90	0.185	0.165	0.167	0.149
	0.93	0.115	0.112	0.106	0.104
	1.00	0.174	0.133	0.174	0.133
	2.57	0.215	0.260	0.553	0.668
	2.76	0.204	0.149	0.563	0.410
	2.88	0.196	0.175	0.563	0.504
	3.33	0.185	0.236	0.614	0.786
	4.79	0.250	0.225	1.198	1.078
	4.95	0.157	0.099	0.775	0.490
	7.31	0.172	0.252	1.257	1.838
					
Mean		0.1861	0.1818		
Standard deviation		0.0343	0.0562		

). To check the reproducibility, the sections were evaluated twice by LD. No significant intraobserver difference (PIMO: *P*=0.4469; CA IX: *P*=0.7425) was seen with an interval between scoring of 1 month. Therefore, the mean of the positive fractions was used in further analyses. Random selected sections were evaluated by a second observer (WL) independently. No significant difference (PIMO: *P*=0.6950; CA IX: *P*=0.1612) was seen. The mean hypoxic fraction, assessed with PIMO and CA IX staining, was 18.6±3.4% and 18.2±5.6%, respectively. The relationship between PIMO-positive fraction and CA IX-positive fraction did not reach statistical significance (*r*=0.5182 and *P*=0.1025).

To allow comparison with the [^18^F]FMISO volume measured with PET, the PIMO-positive and CA IX-positive volume (cm^3^) was calculated by multiplying the MAb staining-positive fraction with the corresponding tumour volume (see Table 1).

### [^18^F]FMISO analysis of normal tissues and tumours

The distribution of [^18^F]FMISO in the rat was determined on the 2 h p.i. images. Although a high [^18^F]FMISO uptake was found in the gastrointestinal region, tumours could be clearly localised.

**Figure 2:**
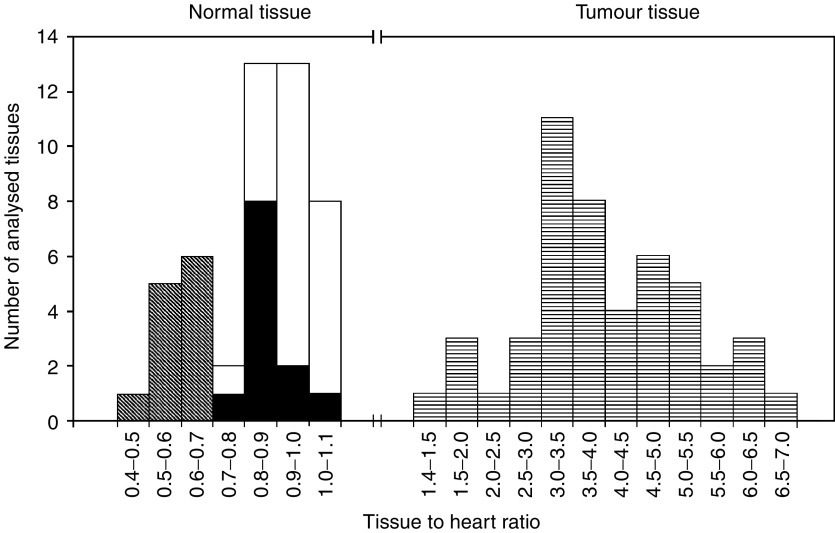
[^18^F]FMISO-PET mean tissue to heart activity ratios of the lung (*n*=12) 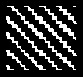, muscle (*n*=24; that is, front leg muscle *n*=12 and hind leg muscle *n*=12) □ and a body area 15 mm above the heart (*n*=12) ▪ on 12 randomly chosen 2 h p.i. images. Similarly, tumours (*n*=48) 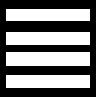 were analysed on 2 h p.i. images. For all the tissues, cumulative histogram analysis was carried out.

The [^18^F]FMISO uptake in normal tissue was evaluated using 2D-ROIs on the lung, muscle and a body area 15 mm above the heart. Figure 2

shows a composite histogram of the calculated tissue to heart ratios. A clear separation of the mean activity ratios is observed at 1.1 between normal and tumour tissue.

**Figure 3:**
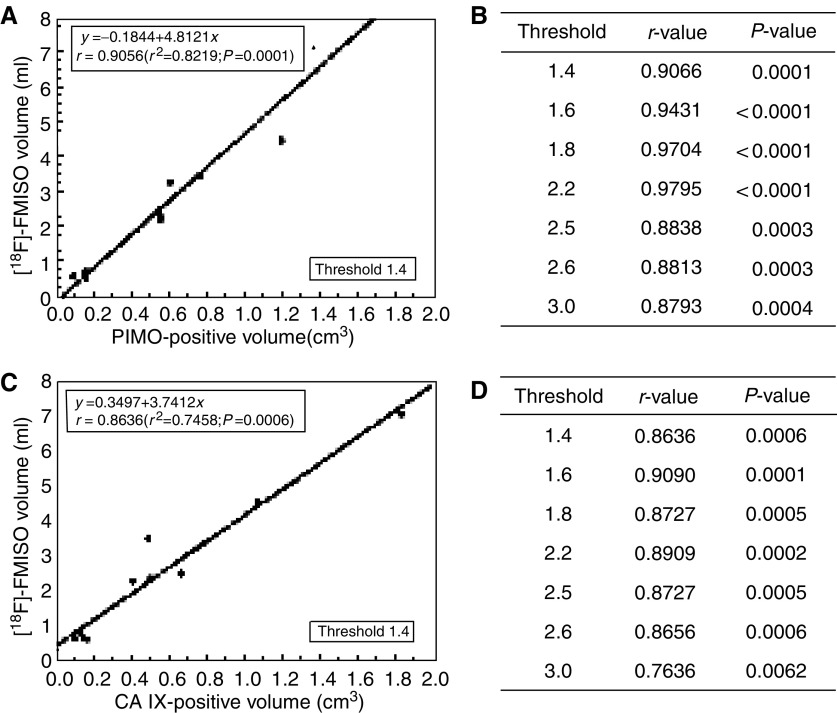
(**A**) [^18^F]FMISO volume defined on 2 h p.i. images, using a threshold 1.4 plotted against PIMO-positive volume (*n*=11 tumours). (**B**) *r*- and *P*-values for a range of thresholds between 1.4 and 3.0 for the same comparison. (**C**) [^18^F]FMISO volume defined on 2 h p.i. images, using a threshold 1.4 plotted against CA IX-positive volume (*n*=11 tumours). (**D**) *r*- and *P*-values for a range of thresholds between 1.4 and 3.0 for the same comparison.

To define the volume of [^18^F]FMISO uptake in the tumours, different thresholds ranging between 1.2 and 3.0 were used. The use of the lowest threshold (1.2) resulted in [^18^F]FMISO volumes that were larger than the calliper-defined tumour volume. The correlation for the various thresholds above 1.4 between the [^18^F]FMISO volume and the PIMO-positive and CA IX-positive volumes is shown in Figure 3

. The *P*-value for each correlation is also given.

### Autoradiography data

**Figure 4:**
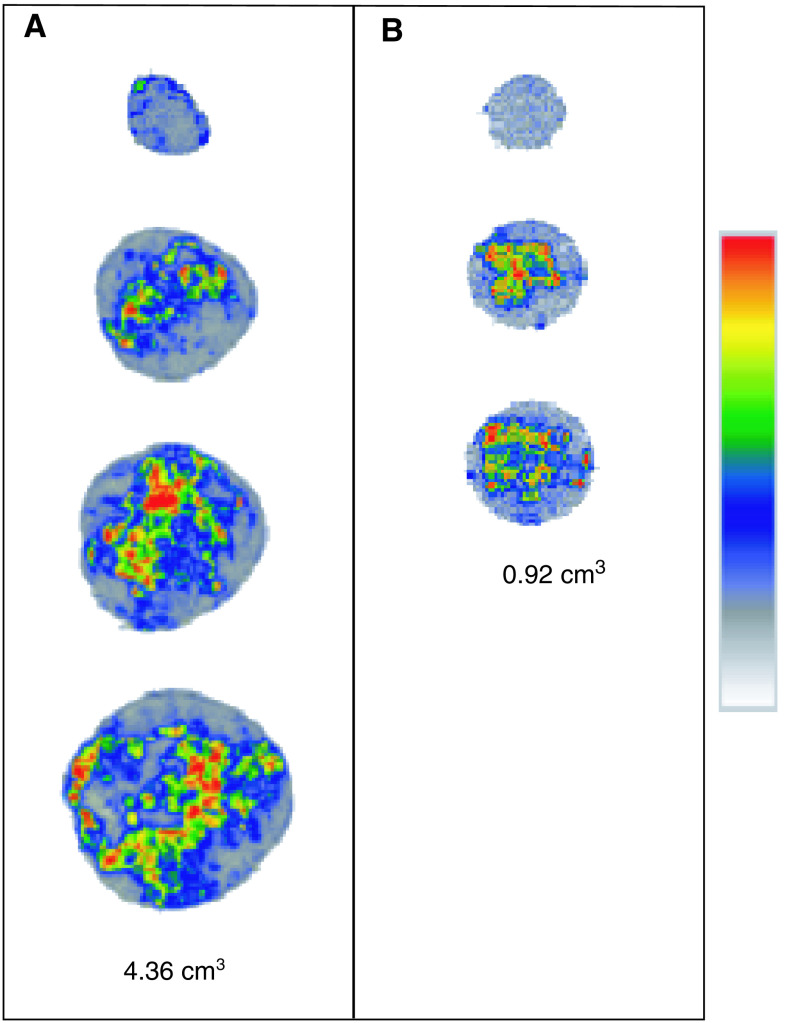
Autoradiography images of a large (**A**: 4.36 cm^3^) and a small (**B**: 0.92 cm^3^) tumour. From the top to bottom of the images, peripheral to central images are shown. Each image is scaled to the hottest pixel (=most [^18^F]FMISO uptake) in the tumour. Images are enlarged to 200%.

[^18^F]FMISO was heterogeneously distributed within tumours. More heterogeneity was, however, seen in the central part of the tumour, showing regions with high uptake and regions with little uptake. In the central part of the tumour, a factor 7 difference was observed between the highest and lowest intensity of [^18^F]FMISO uptake. The lowest intensity of the central part was equal to the mean intensity of the peripheral part of the tumour. This phenomenon was seen in both large tumours (see Figure 4A

) and small tumours (see Figure 4B). For larger tumours, larger areas without [^18^F]FMISO labelling were seen.

## DISCUSSION

The knowledge that hypoxia influences the outcome of many cancer treatment modalities emphasises the necessity to measure these hypoxic cell populations in individual tumours. Comparative studies allow validation of novel methodologies by comparison with established ‘so-called standard’ techniques. Only two comparative studies between noninvasive [^18^F]FMISO-PET and ‘standard’ hypoxia measurements have been published to date. Bentzen *et al* (2002) found no correlation between [^18^F]FMISO-PET and pO_2_ electrode measurements in C3H mammary carcinomas. Piert *et al* (1999), (2000) showed a correlation between [^18^F]FMISO-PET data and pO_2_ electrode measurements in a study of hypoxia in pig liver tissue. Until today, however, the potential of this PET technique still needs confirmation by appropriate procedures, such as comparative evaluation with nitroimidazole-related assays.

In the present study, the noninvasive [^18^F]FMISO-PET method for the evaluation of hypoxia in experimental rat tumours was further validated with immunohistochemical staining techniques using the nitroimidazole PIMO, a ‘standard’ exogenous hypoxia marker, and morphometry. In addition, also CA IX, an endogenous indicator of hypoxia, was used. Microscopy-based point counting, a method used in morphometric tissue analysis (Weibel, 1981) and also in our study, is next to computerised image analysis shown to be an adequate method for quantification of hypoxia in tumours (Varia *et al*, 1998).

[^18^F]FMISO volumes are the direct result of the PET analysis. To compare these data with the immunohistochemical measurements, the staining-positive fractions of PIMO and CA IX were multiplied with the corresponding tumour volumes to obtain the respective hypoxic volumes. The decision to use hypoxic volumes rather than hypoxic fractions is also supported by Rajendran *et al* (2003), who discussed the fact that the use of hypoxic fractions is a variable with considerable uncertainty.

In a range between 1.4 and 2.2, the hypoxic volumes obtained with [^18^F]FMISO-PET correlated to the same high statistical significance with the PIMO-derived hypoxic volumes. A similar observation was made with the CA IX-derived hypoxic volumes. Although only a slight decrease in correlation was calculated, a dropout of data was present at a threshold above 2.2. The choice to use the 2 h p.i. [^18^F]FMISO-PET images was made for the evaluation of the tracer uptake, because this time point has been shown to be optimal for the examination of [^18^F]FMISO uptake in tumours both in animal models (Kubota *et al*, 1999; Tochon-Danguy *et al*, 2002) and in humans (Koh *et al*, 1992; Valk *et al*, 1992). Selection of normal tissue (lung, muscle and a body area 15 mm above the heart) for image analysis at 2 h p.i. using 2D ROIs, was performed in agreement with the literature (Koh *et al*, 1992; Bentzen *et al*, 2000). Mean activity ratios were calculated and 99% of the analysed ratios were below 1.1. Tumour analysis indicated that using thresholds below 1.4 did not seem useful, because results indicated that the [^18^F]FMISO volume was larger than the calliper-defined tumour volume. This can be explained by the spatial resolution of the PET system, where the relatively large voxels, which encompass partial hypoxic regions, are considered as completely hypoxic. Based on these normal and tumour tissue observations, a threshold of 1.4 was defined as cutoff value and as indicator of significant hypoxia. This cutoff value is in agreement with the results of Koh *et al* (1992).

We are aware that within the rhabdomyosarcoma tumour type the hypoxic volumes tend to increase with tumour size. This is however tumour type dependent and we realise therefore that the same comparisons need to be carried out in other tumour models, at best where this relationship does not hold. A positive relationship between the hypoxic volumes assessed with [^18^F]FMISO-PET and PIMO staining was to some extent anticipated. Indeed, both are 2-nitroimidazoles, which have the same nitroreduction mechanism, and are thus expected to bind to intracellular macromolecules in cells exposed to equal microenvironmental hypoxia conditions (Raleigh and Koch, 1990; Casciari *et al*, 1995). The comparison between [^18^F]FMISO-PET and CA IX staining was also investigated, resulting in a similar strong correlation and significance. However, a factor 4 difference was found, which may be explained by the inferior resolution and the large voxel size of the PET system when compared with immunohistochemistry. The extent of a positive correlation between immunohistochemical staining results for these markers is the subject of a controversial discussion. Although Olive *et al* (2001) found a very strong correlation (*r*=0.86, *n*=14) in human cervical cancer, Airley *et al* (2003) did not find a significant correlation (*r*=0.27; *P*=0.083, *n*=42). In head and neck cancer, Kaanders *et al* (2002) found a weak, but significant correlation (*r*=0.36; *P*=0.02, *n*=42). Lyng *et al* (1997), Raleigh *et al* (1999) and Olive *et al* (2000) showed for a number of animal and human tumours that PIMO labelling can give a reliable estimate of radiobiologically relevant hypoxia. The variations in correlation between PIMO- and CA IX-stained hypoxic fractions may be explained by a difference in specificity of CA IX when compared with PIMO (Kaanders *et al*, 2002). A difference in location and area of positive staining (Wykoff *et al*, 2000; Olive *et al*, 2001 and also the present study) supports this explanation. von Hippel-Lindau mutations, which result in an upregulation of HIF-1*α* (Cockman *et al*, 2000) and downstream components like CA IX (Ashida *et al*, 2002), and the relationship between CA IX expression and acidic pH found in tumours (Stubbs *et al*, 2000) could potentially contribute to the published differences.

Autoradiography was performed to determine the [^18^F]FMISO distribution in whole tumours. Different studies have shown heterogeneity in, for example, larger C3H tumours growing subcutaneously in CDF1 mice and a more uniform radioactivity distribution in smaller C3H tumours (Bentzen *et al*, 2002; Grönroos *et al*, 2004). Tochon-Danguy *et al* (2002) observed only homogeneous distribution of [^18^F]FMISO throughout C6 glioma tumours in the left brain of Wistar rats. In the present study, a heterogeneous distribution of [^18^F]FMISO was found both in small and large rhabdomyosarcoma tumours. More heterogeneity was observed in central parts of the tumour compared to peripheral parts. For larger tumours, larger areas with no [^18^F]FMISO labelling were seen, which parallels the fact that larger parts of these tumours are necrotic. Although no direct comparison was made on the same sections with the autoradiography data, immunohistochemical staining with PIMO confirms these results. A heterogeneous stain was seen in all tumours, both in peripheral and central parts of the tumour. A similar heterogeneous staining distribution of hypoxic areas was found in CA IX-stained sections. These autoradiography and immunohistochemical staining results support the concept that the microenvironment of solid tumours is characterised by heterogeneity in oxygenation.

## CONCLUSION

The strong and significant relationship between [^18^F]FMISO-PET and PIMO immunohistochemical staining, within a range of thresholds (1.4–2.2), indicates the value of [^18^F]FMISO-PET to measure hypoxic volumes in whole tumours. Although a positive correlation between PIMO and CA IX is not a general finding, the equally significant and strong correlation between [^18^F]FMISO-PET and CA IX immunohistochemical staining strengthens the application of the noninvasive PET method to evaluate hypoxia. Given the complex nature of hypoxia development and its impact on tumour progression and treatment response, it remains highly important to make additional comparative studies and to relate [^18^F]FMISO measurements with outcome.
